# Diphenylhydantoin (phenytoin)-induced chronic pulmonary disease

**DOI:** 10.4103/0970-2113.56356

**Published:** 2009

**Authors:** Ramakant Dixit, Kalpana Dixit, Paras Nuwal, Arunima Banerjee, Sidharth Sharma, Lokendra Dave

**Affiliations:** *Department of Pulmonary Medicine, J. L. N. Medical College, Ajmer, India*; 1 *Department of Pathology, J. L. N. Medical College, Ajmer, India*; 2 *Department of Surat Municipal Institute of Medical Education & Research (SMIMER), Surat, India*

**Keywords:** Pulmonary disease, diphenylhydantoin/phenytoin, interstitial lung disease

## Abstract

Drug-induced respiratory diseases are difficult to diagnose and therefore usually not identified, probably underestimated and under-reported. We report a case of diphenylhydantoin/phenytoin-induced chronic pulmonary disease in a 62-year-old male patient presenting with progressive dyspnea, eosinophilia, and pulmonary abnormalities. The importance of drug history in clinical history-taking and early diagnosis of drug-induced respiratory diseases is emphasized so as to prevent permanent pulmonary damage.

## INTRODUCTION

Iatrogenic respiratory disease is an important cause of patient morbidity and mortality. Clinical and radiological findings are nonspecific, and diagnosis can be difficult. Therefore, it is important for physicians to be familiar with iatrogenic respiratory disease caused by drugs, transplantation, radiation, transfusion, or other miscellaneous therapies.[1] Unfortunately, drug-induced respiratory disease (DIRD) remains a disease of exclusion; as in the majority of the drugs that are responsible for the disease, this cannot be diagnosed by any specific test. Even pathology may not solve the issue. The diagnosis of DIRD usually is based on several criteria, including[2] (i) a history of drug exposure; (ii) clinical, imaging, and histopathological patterns that are consistent with earlier observations with the same drugs; (iii) exclusions of other lung disease; (iv) improvement following discontinuation of the suspected drugs; and (v) recurrence of symptoms on rechallenges (this may be done in certain circumstances but usually is considered unethical with regard to drugs that cause irreversible pulmonary changes).

A variety of complications of diphenylhydantoin (DPH)/ phenytoin therapy have been described, but there are few reports pertaining to pulmonary changes. The present report describes a case of DPH-induced chronic pulmonary disease presenting as progressive dyspnea, eosinophilia, and pulmonary abnormalities in a 62-year-old male patient.

## CASE REPORT

A 62-year-old man presented with history of progressive breathlessness for the last eight years; and recently, low-grade fever, cough with purulent expectoration, and breathlessness at rest since the last three months. He was treated with various oral antibiotics, but his condition increasingly worsened. He had past history of appendicectomy 20 years back and fracture left humerus 15 years back. He is a known case of epilepsy since the last 15 years, which is controlled by oral diphenylhydantoin (phenytoin) 50 mg daily since the last 11 years. He is a shopkeeper, has never smoked, and has no significant family history. He also has no history of worm infestation; exposure to environmental, agricultural, and chemical pollutants; allergic diathesis or drug hypersensitivity reaction.

On examination, the patient was of average build, moderately nourished, with no anemia, icterus, clubbing, cyanosis, peripheral lymphadenopathy, skin rash, etc. There was nothing abnormal on general physical examination. His pulse rate was 110/min, respiration-38 breaths/min, blood pressure-110/70 mmHg, and temperature-99°F. Examination of respiratory system revealed bilateral diffuse, fine, late inspiratory crackles. Other systemic examinations were unremarkable.

**Figure 1:**
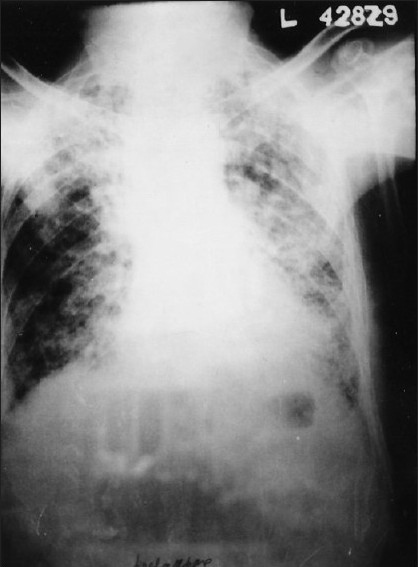
X-ray chest, PA view, showing bilateral inhomogeneous infiltrates, more on the left side

**Figure 2:**
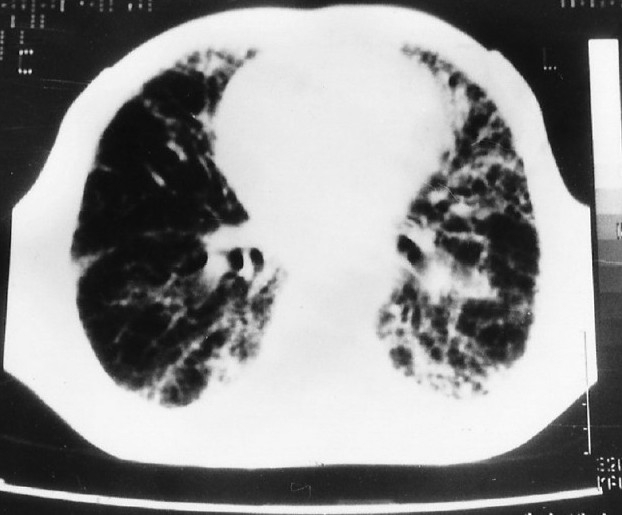
CT scan thorax showing interstitial lung disease pattern

**Figure 3:**
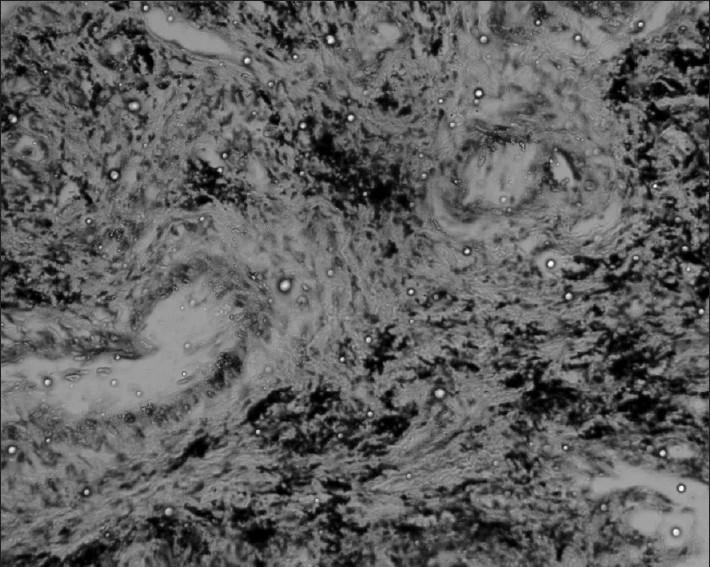
Photomicrograph of transbronchial lung biopsy showing features of chronic interstitial pneumonitis (H and E, ×200)

His hemoglobin was 13 g%, total leukocyte count 12,800/ μL (polymorphs-62%, lymphocytes-20%, and eosinophils-18%), erythrocyte sedimentation rate - 19 mm in the first hour by Wintrobes method, blood sugar - 84 mg/ dL, blood urea - 24 mg/dL, serum creatinine - 0.7 mg/dL, SGOT - 28 IU, serum alkaline phosphatase - 95 IU/L, serum Na^+^ 142 mEq/L, serum K^+^ 4.1 mEq/L, serum Ca^+^ 10.5 mg/dL, with normal urine analysis, etc. Skiagram chest, PA view, revealed bilateral inhomogeneous infiltrates (more on the left side) with mild cardiomegaly [Figure 1]. His ECG was normal, but echocardiography revealed mild mitral regurgitation (MR), mild tricuspid regurgitation (TR), ejection fraction of 50%, normal left ventricle size and systolic function with reduced compliance. Results of serological tests for HIV, HBsAg, ANA, and ANCA were negative. The filarial and Aspergillus serological findings were also negative. Findings from sputum smears and culture examinations for pathogenic organisms, mycobacteria, and fungi were also negative. Stool examination gave a negative result for parasitic infestation. His arterial blood gas analysis revealed pH - 7.38, pO_2_ - 57.4, pCO_2_ - 41.1, HCO_3_ - 24.4, oxygen saturation- 89.1%, TCO_2_ - 25.7, base excess - 0.5, and shunt - 26.1. His spirometry revealed severe ventilatory abnormality, with FVC - 1.04 L (34% of the predicted), FEV_1_ - 0.91 L (37% of the predicted), FEV_1_/FVC ratio - 87%, PEF - 2.12 L/s (28% of the predictive value), and FEF_25-75_ - 0.65 L/s (19% of the predicted), etc. CT scan thorax revealed bilateral coarse interlobular septal thickening with perilymphatic nodularity and focal areas of reticular pattern more prominent at the lung periphery and bases [Figure 2]. There was mild pleural thickening at apex, but there was no evidence of pleural effusion, mediastinal lymphadenopathy, etc. Fiber-optic bronchoscopy revealed no endobronchial abnormalities, and bronchoalveolar lavage analysis revealed mixed inflammatory cells, including eosinophils, with no malignant cells, or cells with abnormal morphology, organisms (i.e., bacteria, acid-fast bacilli, fungi, and larva), or dust particles, etc. Transbronchial lung biopsy showed predominant chronic interstitial pneumonitis pattern with fibroblast proliferation, alveolar septal thickening, partial loss of alveolar architecture, and alveolar septal lympho-plasmocytic and eosinophilic infiltrates [Figure 3]. No granulomatous component or vasculitis or necrosis was evident.

**Figure 4:**
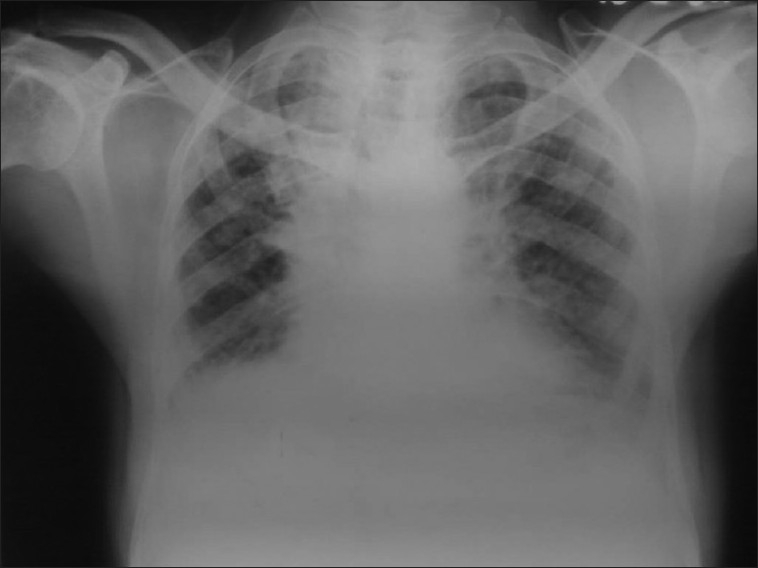
X-ray chest, PA view, after phenytoin withdrawal, showing marked radiological improvement

The patient was initially managed with oxygen therapy, broad-spectrum antibiotics, and bronchodilators, along with supportive therapy. Fever and expectoration of sputum declined with the above-mentioned line of management, but breathlessness and peripheral eosinophilia persisted and even did not get corrected with a therapeutic trial of diethylcarbamizine therapy. The changes were felt to be related to DPH therapy. Lymphocyte stimulation test with phenytoin was done and found to be positive, with a stimulation index of 292%. Previous phenytoin therapy was stopped, and oral prednisolone (1 mg/Kg body wt/day) was added immediately, which resulted in improvements in both eosinophilia and, to some extent, breathlessness after two weeks. The patient was discharged after six weeks in a stable condition with partially improved lung functions but lost to follow-up for the next five months. Thereafter, he came for routine checkup, and this time he had breathlessness only on exertion with improvement in lung functions revealed on spirometry and marked clearance of radiographic abnormalities [Figure 4]. He also documented that he did not take any medications, including phenytoin or steroids, during that period.

## DISCUSSION

Our patient presented with features of chronic interstitial lung disease, peripheral eosinophilia, and chronic interstitial pneumonitis on histological examination, with no evidence of allergic, parasitic, vasculitic, neoplastic, or other known cause of eosinophilia. He was on a single drug, viz., phenytoin, before the onset of symptoms, and there was nothing to suggest any other primary or secondary pulmonary disease. A history of drug intake with a temporal association raised the diagnosis of DIRD caused by phenytoin in our case (as per the criteria mentioned in the literature[3]); and lymphocyte-stimulation test with phenytoin further supported the diagnosis. More so, the clinical and radiological abnormalities significantly improved after phenytoin withdrawal.

More than 110 drugs are reported to cause drug-induced eosinophilia, and common among them include amidarone, bleomycin, captopril, gold salts, iodine, radiographic contrast media, L-tryptophan, methotrexate, nitrofurantoin, etc., apart from phenytoin.[1,4,5]

Histological findings resembling chronic interstitial lung disease are commonly seen with cytotoxic, antimicrobial, anti-inflammatory, antiarrhythmic, antihypertensive, and anticonvulsant agents, apart from other miscellaneous drugs.[3] Bronchoalveolar lavage (BAL) findings are not specific for any DIRD; however, they contribute to the expected clinico-pathologic pattern of a given DIRD, apart from excluding an infective cause and involvement of lung by underlying disease, i.e., malignancy.[6]

DPH is primarily used in the treatment of seizure disorders and neuralgia and is thought to have therapeutic effect through stabilization of neuronal membranes. Extrapulmonary complications of DPH therapy are well known, but pulmonary complications are debated for long times. Clinical pulmonary disease caused by DPH is rare. On the other hand, subclinical disease caused by DPH is suggested to be more common.[7] Moore[8] reported roentgenographic abnormalities in 87% of patients receiving phenytoin for at least two years, while Hazlett and co-workers[9] found abnormal pulmonary functions in 45% of patients receiving the drug for two or more years but without any symptoms or chest roentgenographic abnormalities. Livingston *et al.,*[10] found no abnormality in pulmonary functions among 43 patients but minimal radiological changes in two patients receiving the drug for at least two years. Acute complications following phenytoin therapy are also described. Acute pulmonary disease caused by DPH presents with fever, cough, dyspnea, hypoxemia, and bilateral radiographic infiltrates, which appears reversible with cessation of the drug and treatment with corticosteroids.[11] DPH therapy has occasionally been reported to cause hypersensitivity lung disease in some cases.[12]

Histopathological changes in lungs during DPH therapy have been described in only few patients. These include interstitial pneumonitis,[11] necrotizing vasculitis[13] and lymphocytic interstitial pneumonitis,[14] etc. The case presented here showed not only abnormal pulmonary functions but radiological infiltrates also, along with features of chronic interstitial pneumonitis on tissue examination; further elucidating the nature of DPH-induced pulmonary changes. Although old literature does not strongly support chronic pulmonary illness caused by DPH,[15] there are subsequent evidences to suggest that DPH can also cause chronic pulmonary changes.[7] Our case also supports the view that DPH can cause chronic pulmonary disease that may be associated with eosinophilia.

We conclude with the remark that busy clinicians should be more vigilant regarding DIRD, as the field of DIRD is expanding at a quick pace and many patients do not tell, unintentionally, their physicians regarding drugs they are taking due to some or the other reason. This needs improvement in recording the drug history in every case and up-to-date knowledge of drugs that can cause DIRD. The website ‘www.pneumotox.com’ provides useful information about DIRD and can therefore be assessed for the same. Some cases of drug-induced eosinophilia more closely resemble the severe and prolonged illness of chronic eosinophilic pneumonia, and therapy with steroids is often employed to hasten resolution.[16] This was true in our case also. An early diagnosis of DIRD is essential as irreversible changes may occur in chronic cases and timely stoppage of the provoking agent can prevent further progression of the disease.

## References

[1] Foucher P, Camus P Pneumotox.

[2] Camus P, Schwarg MI, King TE (2003). Drug induced infiltrative lung disease. Interstitial lung disease.

[3] Flieder DB, Travis WD (2004). Pathologic characteristics of drug induced lung disease. Clin Chest Med.

[4] Allen JN (2004). Drug induced eosinophilic lung disease. Clin Chest Med.

[5] Mahatma M, Haponic EF, Nelson S, Lopez A, Summer WR (1989). Phenytoin induced acute respiratory failure with pulmonary eosinophilia. Am J Med.

[6] Costabel U, Uzaslan E, Guzman J (2004). Bronchoalveolar lavage in drug induced lung disease. Clin Chest Med.

[7] Cooper J, Allen D, White DA, Matthay RH (1986). Drug induced pulmonary disease: Part 2: Non cytotoxic drugs. Am Rev Respir Dis.

[8] Moore MT (1959). Pulmonary changes in hydantoin therapy. JAMA.

[9] Hazlett DR, Ward GW, Madison DS (1974). Pulmonary function loss in diphenylhydantoin therapy. Chest.

[10] Livingston S, Whitehouse D, Pauli L (1961). Study of the effects of diphenyl-hydantoin sodium on the lungs. N Engl J Med.

[11] Michael JR, Rudin ML (1981). Acute pulmonary disease caused by phenytoin. Ann Intern Med.

[12] Fruchter L, Laptook A (1981). Diphenylhydantoin hypersensitivity reaction associated with interstitial pulmonary infiltrates and hypereosinophilia. Ann Allergy.

[13] Yermakov VM, Hitti IF, Sutton AL (1983). Necrotising vasculitis associated with Diphenylhydantoin: Two fatal cases. Hum Pathol.

[14] Chamberlain DW, Hyland RH, Ross DJ (1986). Diphenylhydantoin induced lymphocytic interstitial pneumonia. Chest.

[15] Low NL, Yahr MD (1960). The lack of pulmonary fibrosis in patients receiving diphenylhydantoin. JAMA.

[16] Leitch AG, Seaton A, Seaton D, Leitch AG (2002). Pulmonary eosinophilia. Crofton and Douglas's respiratory diseases.

